# ﻿New species of *Aspergillus* in sections *Cavernicolarum* and *Nigri* from terrestrial ecosystems of China (Eurotiales, Aspergillaceae)

**DOI:** 10.3897/mycokeys.124.172775

**Published:** 2025-11-03

**Authors:** Lu-Yao Peng, Wen-Ying Zhuang, Xin-Cun Wang

**Affiliations:** 1 State Key Laboratory of Microbial Diversity and Innovative Utilization, Institute of Microbiology, Chinese Academy of Sciences, Beijing 100101, China Institute of Microbiology, Chinese Academy of Sciences Beijing China; 2 University of Chinese Academy of Sciences, Beijing 100049, China University of Chinese Academy of Sciences Beijing China

**Keywords:** Aspergillaceae, biodiversity, Eurotiales, phylogeny, taxonomy

## Abstract

*Aspergillus* species are of great industrial, agricultural, and medicinal importance. During investigations on the biodiversity of Aspergillaceae, two species of *Aspergillus* isolated from soil samples in China were identified as new to science based on sequence analyses and morphological comparisons. *Aspergillus hebeiensis* from a traditional cultural and Buddhist heritage site is the second member of the series Hainanici in section Cavernicolarum of subgenus Nidulantes, while *A. xishuangbannaensis* from a tropical nature reserve is classified in subgenus Circumdati, section Nigri, series *Japonici*. Detailed descriptions and illustrations of both species are provided, and the ecological functions of their habitats are also discussed.

## ﻿Introduction

Species of *Aspergillus* P. Micheli ex Haller are ubiquitous in various environments and have a long history of exploitation by humans. Some species have been used for food fermentations for centuries, especially in East Asia. *Aspergillus oryzae* (Ahlb.) Cohn and *A. sojae* Sakag. & K. Yamada ex Murak. play crucial roles in the production of rice wine, soybean pastes, and soy sauce (Bennett 2009), and *A. niger* Tiegh. is used for fermentations of baijiu (Xu et al. 2022) as well as pu-erh tea (Frisvad et al. 2007). More than 100 years ago, the ability of *A. niger* to produce citric acid was industrially exploited in 1919 (Schuster et al. 2002). *Aspergillus terreus* Thom was the first of the major statins to produce lovastatin, a medicine used to lower cholesterol (Bennett 2009). *Aspergillus niger* and *A. terreus* are also efficient agents for bioleaching of rare earth elements with low environmental impact (Mowafy 2020). *Aspergillus cvjetkovicii* Jurjević et al. can protect against phytopathogens through interspecies chemical signaling in the phyllosphere of rice as a biocontrol agent (Fan et al. 2024). On the other hand, some species pose severe threats to human health. Mycotoxins are produced by certain *Aspergillus* species, causing food contamination, e.g., aflatoxins by *A. flavus* Link and *A. parasiticus* Speare, and ochratoxin A by *A. ochraceus* G. Wilh. and *A. niger* (Xue et al. 2025). *Aspergillus fumigatus* Fresen. is known as the primary causative agent of aspergillosis, followed by *A. flavus*, *A. niger*, and *A. terreus* (Khan et al. 2024).

The genus *Aspergillus* was originally introduced in 1729 and validated in 1768. It was divided into six subgenera, 27 sections, and 75 series, with 446 species recognized (Houbraken et al. 2020). A new series, Aspergillus ser. Hainanici, was recently proposed (Wang and Zhuang 2022), and the accepted species of the genus increased to 453 (Visagie et al. 2024). Subsequently, 16 newly described species were added: one each from Europe and North America, two from South America, and the remaining 12 from Asia. *Aspergillus albicolor* D.S. Paiva was reported from Portugal, *A. pseudoalabamensis* Cañete-Gibas et al. from the USA, and *A. alvaroi* J.M.S. Lima et al. and *A. guanovespertilionum* J.M.S. Lima et al. from Brazil. Among the 12 Asian taxa, five were from China (*A. cylindricus* Zhi Y. Zhang et al., *A. doliiformis* Zhi Y. Zhang et al., *A. liaoningensis* C. Liu et al., *A. plumeriae* C. Liu et al., *A. subinflatus* C. Liu et al.), *A. dhakephalkarii* Rajeshk. et al. and *A. patriciawiltshireae* Rajeshk. et al. from India, *A. hubkae* Y.B. Zhou et al. and *A. mahabadiensis* Abdollahz. & O. Ghaderi from Iran, *A. verrucosus* R. Hagiuda & D. Hirose from Japan, *A. ullungdoensis* Hyang B. Lee from South Korea, and *A. halopiscium* V.N. Thanh et al. from Vietnam.

During investigations on the biodiversity of Aspergillaceae in China, two species of *Aspergillus* isolated from soil were identified as new to science based on sequence analyses and morphological comparisons. Detailed descriptions and illustrations are provided.

## ﻿Materials and methods

### ﻿Fungal materials

Cultures were isolated from soil samples collected from Hebei and Yunnan provinces, China. Dried cultures were preserved in the Herbarium Mycologicum Academiae Sinicae (HMAS, Beijing, China), and the living ex-type strains were deposited in the China General Microbiological Culture Collection Center (CGMCC, Beijing, China).

### ﻿Morphological observations

Morphological characteristics were observed and recorded according to standardized methods (Samson et al. 2014). Four standard growth media were adopted: Czapek yeast autolysate agar (CYA; yeast extract, Oxoid, Hampshire, UK), malt extract agar (MEA; Amresco, Solon, OH, USA), yeast extract agar (YES), and potato dextrose agar (PDA). The methods for colonial inoculation, incubation, macroscopic and microscopic examinations, and digital capture followed our previous studies (Wang and Zhuang 2022; Wang et al. 2023; Peng et al. 2025).

### ﻿DNA extraction, PCR amplification, and sequencing

DNA was extracted from living cultures grown on PDA for 7 days using the Plant Genomic DNA Kit (DP305; TIANGEN Biotech, Beijing, China). Polymerase chain reaction (PCR) amplifications of internal transcribed spacer (ITS), beta-tubulin (BenA), calmodulin (CaM), and RNA polymerase II second-largest subunit (RPB2) were conducted using routine methods (Samson et al. 2014). The forward and reverse primers used for each locus were as follows: ITS5 (5’-GGA AGT AAA AGT CGT AAC AAG G-3’) and ITS4 (5’-TCC TCC GCT TAT TGA TAT GC-3’) for ITS (White et al. 1990); Bt2a (5’-GGT AAC CAA ATC GGT GCT GCT TTC-3’) and Bt2b (5’-ACC CTC AGT GTA GTG ACC CTT GGC-3’) for BenA (Glass and Donaldson 1995); CMD5 (5’-CCG AGT ACA AGG ARG CCT TC-3’) and CMD6 (5’-CCG ATR GAG GTC ATR ACG TGG-3’) for CaM (Hong et al. 2005); and 5F (5’-GAY GAY MGW GAT CAY TTY GG-3’) and 7CR (5’-CCC ATR GCT TGY TTR CCC AT-3’) for RPB2 (Liu et al. 1999). The products were sequenced on an ABI 3730 DNA Sequencer (Applied Biosystems, Foster City, CA, USA).

### ﻿Phylogenetic analyses

The newly generated forward and reverse sequences in this study were assembled using SeqMan v. 7.1.0 (DNASTAR Inc., Madison, WI, USA). The assembled sequences were deposited in GenBank, with accession numbers shown in bold (Tables 1, 2). The additional sequences used for phylogenetic analyses are also listed. Sequences were aligned using MAFFT v. 7.221 (Katoh and Standley 2013), either as individual single-gene datasets (ITS, BenA, CaM, and RPB2) or concatenated datasets. They were then manually edited and concatenated in BioEdit v. 7.1.10 (Hall 1999) and MEGA v. 11.0.13 (Tamura et al. 2021). Maximum likelihood (ML) analyses were performed using the IQ-TREE web server (Trifinopoulos et al. 2016) with the default automatic substitution model and bootstrap (BP) iteration (1,000 replicates) settings. Bayesian inference (BI) analyses were conducted with MrBayes v. 3.2.7 (Ronquist et al. 2012). Modeltest v. 3.7 (Posada and Crandall 1998) was adopted to determine appropriate nucleotide substitution models and parameters. Four MCMC chains (three heated and one cold) were run for at least 1 million generations, and posterior probability (PP) values were calculated based on the remaining 75% of trees after the burn-in phase. The consensus trees were viewed using FigTree v. 1.4.4 (http://tree.bio.ed.ac.uk/software/figtree, accessed on 28 December 2023).

**Table 1.** Species and sequences used in the phylogenetic analyses for Aspergillus subgenus Nidulantes section Cavernicolarum.

Series	Species	Strain	Locality	Substrate	ITS	BenA	CaM	RPB2
*** Cavernicolarum ***	*A. californicus* Frisvad et al., 2011	CBS 123895 T	USA	chaparral of *Adenostoma fasciculatum*	FJ531153	FJ531180	FJ531128	MN969065
	*A. cavernicola* Lörinczi, 1969	CBS 117.76 T	Romania	on walls of cave	EF652508	EF652332	EF652420	EF652244
	*A. kassunensis* Baghd., 1968	CBS 419.69 T	Syria	soil	EF652461	EF652285	EF652373	EF652197
	*A. subsessilis* Raper & Fennell, 1965	CBS 502.65 T	USA	desert soil	EF652485	EF652309	EF652397	EF652221
*** Egyptiaci ***	*A. egyptiacus* Moub. & Mustafa, 1972	CBS 656.73 T	Egypt	sandy soil	EF652504	EF652328	EF652416	EF652240
*** Hainanici ***	*A. hainanicus* X.C. Wang & W.Y. Zhuang, 2022	CGMCC 3.20888 T	China: Hainan	sandy soil	OM414846	OM475626	OM475630	OM475634
	***A. hebeiensis* X.C. Wang, L.Y. Peng & W.Y. Zhuang, sp. nov.**	JJJ40-31 T	China: Hebei	soil under *Platycladus orientalis*	**PV883250**	**PV877068**	**PV877070**	**PV877073**
		JJJ40-12	China: Hebei	soil under *Platycladus orientalis*	**PV883251**	n.a.	**PV877071**	**PV877074**

GenBank accession numbers in bold indicate the newly generated sequences. “n.a.” is the abbreviation for “not available.”

**Table 2.** Species and sequences used in the phylogenetic analyses for Aspergillus subgenus Circumdati section Nigri.

Series	Species	Strain	Locality	Substrate	ITS	BenA	CaM	RPB2
*** Japonici ***	*A. aculeatinus* Noonim et al., 2008	CBS 121060 T	Thailand	dried parchment and green beans of *Coffea arabica*	EU159211	EU159220	EU159241	HF559233
	*A. aculeatus* Iizuka, 1953	CBS 172.66 T	Japan	unknown	EF661221	HE577806	EF661148	EF661046
	*A. brunneoviolaceus* Bat. & H. Maia, 1955	CBS 621.78 T	Brazil	gills of Osteichthyes	AJ280003	EF661105	EF661147	EF661045
	*A. dhakephalkarii* Rajeshk. et al., 2025	NFCCI 5750 T	India	rhizosphere soil associated with *Anthurium andraeanum*	PP741453	PP739067	PP739063	PP739059
	*A. floridensis* Jurjevic et al., 2012	NRRL 62478 T	USA	air	MN431366	HE984412	HE984429	HE984376
	*A. hydei* Doilom, 2020	KUMCC 18-0196 T	China: Yunnan	air under *Quercus variabilis*	MT152332	MT161679	MT178247	MT384370
	*A. indologenus* Frisvad et al., 2011	CBS 114.80 T	India	soil	AJ280005	AY585539	AM419750	HE984366
	*A. japonicus* Saito, 1906	CBS 114.51 T	unknown	unknown	AJ279985	HE577804	FN594551	MN969079
	*A. labruscus* Fungaro et al., 2017	IBT 33586 T	Brazil	fruit of *Vitis labrusca*	KU708544	KT986014	KT986008	MN969196
	*A. oxumiae* C.N. Figueiredo et al., 2020	CCDCA 11546 T	Brazil	soil cultivated with *Agave sisalana*	MN431160	n.a.	MN531842	MN521389
	*A. patriciawiltshireae* Rajeshk. et al., 2025	NFCCI 5959 T	India	soil	PQ826401	PQ855384	PQ855382	PQ855386
	*A. saccharolyticus* A. Sørensen et al., 2011	CBS 127449 T	Denmark	toilet seat of treated *Quercus* wood	HM853552	HM853553	HM853554	HF559235
	*A. serratalhadensis* L.F. Oliveira et al., 2018	URM 7866 T	Brazil	soil	MH169127	LT993222	LT993223	LT995971
	*A. trinidadensis* Jurjevic et al., 2012	NRRL 62479 T	Trinidad-Tobago	air	MN431380	HE984420	HE984434	HE984379
	*A. uvarum* G. Perrone et al., 2008	CBS 121591 T	Italy	grape berries	AM745757	AM745751	AM745755	HE984370
	***A. xishuangbannaensis* X.C. Wang, L.Y. Peng & W.Y. Zhuang, sp. nov.**	ZYN05-01 T	China: Yunnan	soil in limestone seasonal rainforest	**PV883252**	**PV877069**	**PV877072**	**PV877075**
*** Nigri ***	*A. niger* Tiegh., 1867	CBS 554.65 T	France	unknown	EF661186	EF661089	EF661154	EF661058

GenBank accession numbers in bold indicate the newly generated sequences. “n.a.” is the abbreviation for “not available.”

## ﻿Results

To determine the species identities of the investigated strains, the single-gene datasets (ITS, BenA, CaM, and RPB2) and the concatenated three-locus (BenA + CaM + RPB2) dataset were compiled and analyzed. The detailed characteristics of the datasets are listed in Table 3.

**Table 3.** Detailed characteristics of the datasets.

Dataset	Gene fragment	No. of seq.	Length of alignment (bp)	No. of variable sites	No. of parsimony-informative sites	Model for BI
*** Cavernicolarum ***	ITS	8	548	64	44	
	BenA	7	471	150	83	
	CaM	8	553	204	129	
	RPB2	8	1071	241	151	
	BenA+CaM+RPB2	8	2095	595	363	TIMef+I
*** Japonici ***	BenA	16	530	235	108	
	CaM	17	580	245	128	
	RPB2	17	1052	273	155	
	BenA+CaM+RPB2	17	2162	753	391	TrN+I+G

Abbreviations of the model: TIMef+I (equal-frequency transition model with invariant sites), TrN+I+G (Tamura–Nei model with invariant sites and gamma distribution).

**Figure 1.**
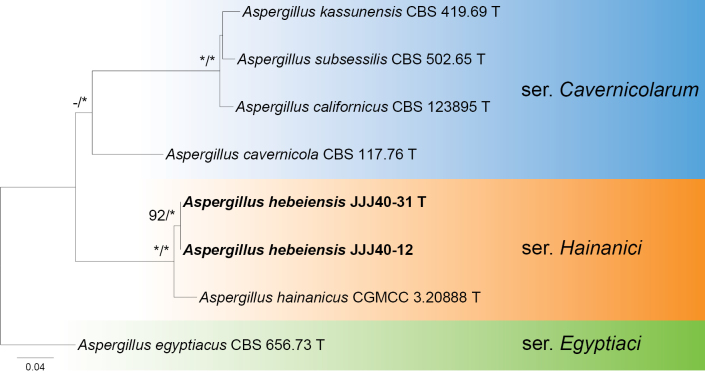
Maximum likelihood phylogeny of Aspergillus subgenus Nidulantes section Cavernicolarum inferred from the combined BenA, CaM, and RPB2 dataset. Bootstrap values ≥ 70% (left) or posterior probability values ≥ 0.95 (right) are indicated at nodes. Asterisks denote 100% bootstrap or 1.00 posterior probability.

In the phylogeny of section Cavernicolarum (Fig. 1), the strains JJJ40-12 and JJJ40-31, representing the same species, were located in series *Hainanici* as sister to *Aspergillus hainanicus*. The close relationship between these two species was strongly supported by statistical values, inferred either from the concatenated dataset (MLBP = 100, BIPP = 1.00; Fig. 1) or from the single-gene datasets (MLBP = 100; Suppl. material 1: figs S1–S4).

**Figure 2.**
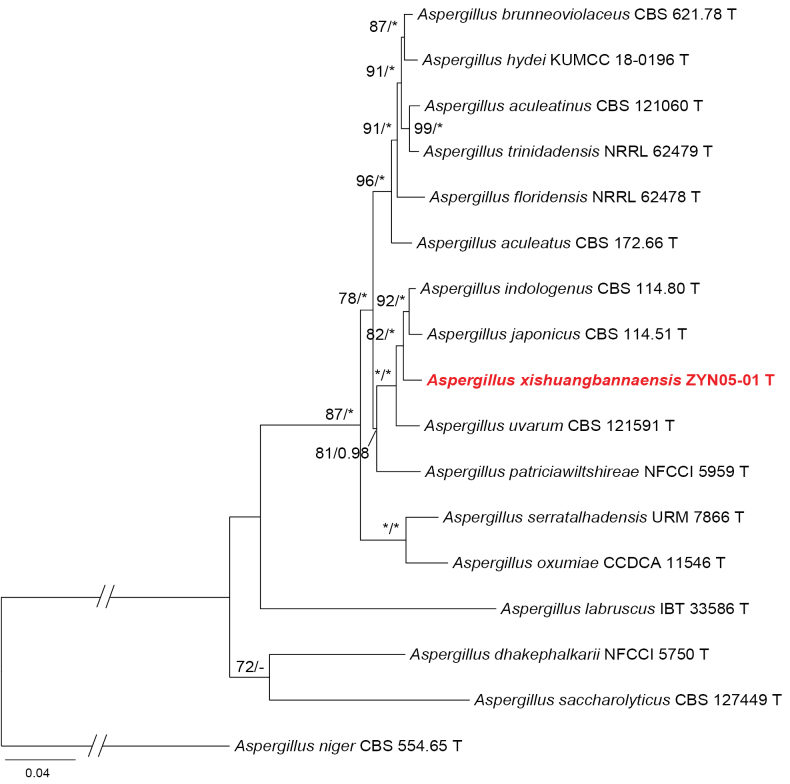
Maximum likelihood phylogeny of Aspergillus subgenus Circumdati section Nigri series *Japonici* inferred from the combined BenA, CaM, and RPB2 dataset. Bootstrap values ≥ 70% (left) or posterior probability values ≥ 0.95 (right) are indicated at nodes. Asterisks denote 100% bootstrap or 1.00 posterior probability.

In the phylogenetic tree of section Nigri series *Japonici* (Fig. 2), strain ZYN05-01 was grouped into a small clade associated with the following species: *A. japonicus*, *A. indologenus*, and *A. uvarum*. A similar tree topology was shown in the BenA analysis (Suppl. material 1: fig. S5), but it differed somewhat from the other two single-gene inferences. Strain ZYN05-01 clustered with *A. uvarum* in the CaM tree (MLBP = 82; Suppl. material 1: fig. S6), but it was closely related to *A. japonicus* in the RPB2 phylogeny (MLBP = 94; Suppl. material 1: fig. S7).

### ﻿Taxonomy

#### ﻿ Aspergillus hebeiensis

Taxon classificationFungiEurotialesAspergillaceae

X.C. Wang, L.Y. Peng & W.Y. Zhuang sp. nov.

55DC9DF9-D078-54D2-9A92-AAFA25F05FD1

Fungal Names: FN 572829

Fig. 3

##### Etymology.

The specific epithet refers to the type locality.

In Aspergillus subgenus Nidulantes section Cavernicolarum series *Hainanici*.

##### Typification.

China • Hebei Province, Handan City, Fengfeng Mining District, Xiangtangshan Caves, one of the First Batch of Key Cultural Relics Units under National Protection of China, Northern Xiangtangshan, 36°32'2"N, 114°9'40"E, in soil under *Platycladus orientalis* (L.) Franco, 17 July 2023, Xin-Cun Wang, culture, Lu-Yao Peng, JJJ40-31 (holotype HMAS 354080, ex-type strain CGMCC 3.29151).

**Figure 3.**
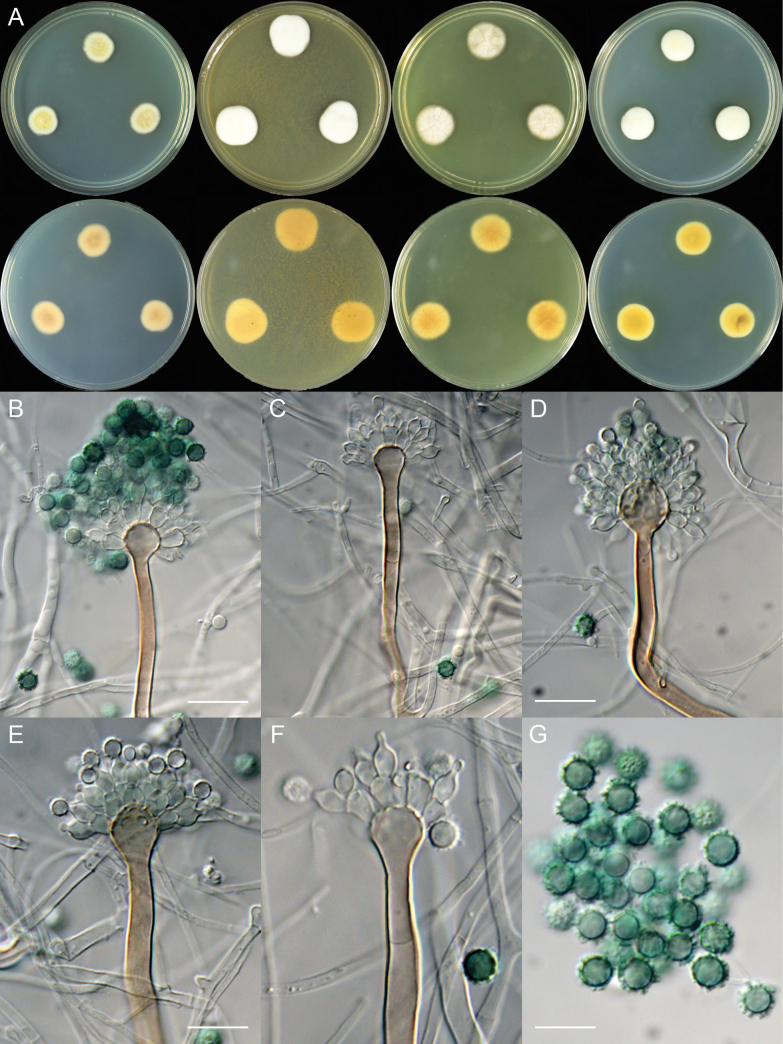
*Aspergillus hebeiensis* (JJJ40-31). A. Colonies: top row left to right, obverse CYA, MEA, YES, and PDA; bottom row left to right, reverse CYA, MEA, YES, and PDA; B–F. Conidiophores; G. Conidia. Scale bars: 17.5 µm (B, C); 15 µm (D); 12.5 µm (E); 10 µm (G, F).

##### DNA barcodes.

ITSPV883250, BenAPV877068, CaMPV877070, RPB2PV877073.

##### Colony diam.

7 days, 25 °C (unless stated otherwise): CYA 15–17 mm; CYA 37 °C 12–15 mm; MEA 19–20 mm; YES 18–19 mm; PDA 16–17 mm.

##### Colony characteristics.

On CYA 25 °C, 7 days: Colonies nearly circular, slightly concave at centers; margins moderately wide, entire; mycelia white and then yellow; texture velutinous; sporulation sparse; conidia *en masse* greyish; soluble pigments absent; exudates absent; reverse buff to yellow brown.

On CYA 37 °C, 7 days: Colonies nearly circular or irregular, slightly protuberant at centers; margins narrow to moderately wide, fimbriate; mycelia white and then buff; texture velutinous; sporulation sparse; conidia *en masse* brownish; soluble pigments absent; exudates absent; reverse buff to yellow brown.

On MEA 25 °C, 7 days: Colonies irregular, protuberant; margins narrow, entire; mycelia white and then cream; texture velutinous; sporulation sparse; conidia *en masse* creamish; soluble pigments absent; exudates absent; reverse buff to yellow brown.

On YES 25 °C, 7 days: Colonies nearly circular, concave at centers, radially sulcate; margins narrow, entire; mycelia pale; texture velutinous; sporulation sparse; conidia *en masse* greyish; soluble pigments absent; exudates absent; reverse yellow brown to orange brown.

On PDA 25 °C, 7 days: Colonies nearly circular, protuberant; margins narrow, entire; mycelia white and then cream; texture velutinous; sporulation sparse; conidia *en masse* creamish to brownish; soluble pigments yellow; exudates absent; reverse buff to yellow brown, occasionally with dark brown sectors.

##### Micromorphology.

Conidial heads radiate; stipes short, 65–110 (–140) × 4.0–7.0 µm, not septate, walls thick, smooth, brown; vesicles 8.5–13 × 8.5–13 µm, subglobose to globose; biseriate; metulae 5.0–8.5 × 3.5–6.5 µm, cylindrical to obovate, covering almost a half to two-thirds surface of the vesicle; phialides 5.5–8.0 × 3.5–4.0 µm, flask-shaped; conidia 6.0–7.5 µm, subglobose, vivid green, strongly echinulate.

##### Additional strain examined.

China • Hebei Province, Handan City, Fengfeng Mining District, Xiangtangshan Caves, one of the First Batch of Key Cultural Relics Units under National Protection of China, Northern Xiangtangshan, 36°32'2"N, 114°9'40"E, in soil under *Platycladus orientalis* (L.) Franco, 17 July 2023, Xin-Cun Wang, culture, Yi-Fan Wang, JJJ40-12.

##### Notes.

This species is the second member of series *Hainanici* and sister to *A. hainanicus* (Fig. 1; Suppl. material 1: figs S1–S4). It differs from the latter by 29 bp for BenA (93.76% sequence identity), 16 bp for CaM (97.03%), and 21 bp for RPB2 (98.04%). Morphologically, although both species have short stipes, biseriate conidiophores, and strongly echinulate conidia, the new species is easily distinguished from *A. hainanicus* by growth on CYA at 37 °C, vivid green, and smaller conidia (6.0–7.5 vs. 6.0–9.5 µm, Table 4).

**Table 4.** Morphological comparisons of the new species and their closely related species.

Series	Species	CYA 25 °C (mm)	CYA 37 °C (mm)	MEA (mm)	YES (mm)	Vesicles (µm)	Conidia shape	Conidia color	Conidia wall	Conidia size (µm)	Reference
*** Hainanici ***	***A. hebeiensis***	15–17	12–15	19–20	18–19	8.5–13 × 8.5–13	subglobose	vivid green	strongly echinulate	6.0–7.5	This study
	*A. hainanicus*	18–20	no growth	16–17	21–22	7.5–13 × 9.0–13	subglobose	hyaline	strongly echinulate	6.0–9.5	Wang and Zhuang 2022
*** Japonici ***	***A. xishuangbannaensis***	64–70	35–57	55–60	68–70	62–80 × 60.5–77.5	subglobose	brown	echinulate	4.5–5.0 × 4.0–5.0	This study
	*A. japonicus*	60–70	20–50	60–70	n.a.	20–35	globose to subglobose	brown	echinulate	3.5–5.0	Klich 2002; Samson et al. 2007
	*A. uvarum*	90	16–22	90	n.a.	20–30	globose to subglobose	brown to black	conspicuously spinose	3.0–4.0	Perrone et al. 2008
	*A. indologenus*	63–70	n.a.	57–70	76–80	20–45	globose	brown	coarsely roughened to echinulate	3.0–4.0	Varga et al. 2011

‘n.a.’ is the abbreviation of ‘not available.’

#### ﻿ Aspergillus xishuangbannaensis

Taxon classificationFungiEurotialesAspergillaceae

X.C. Wang, L.Y. Peng & W.Y. Zhuang sp. nov.

7BF0D4D4-54A3-526F-A181-CEFF4E455AB4

Fungal Names: FN 572923

Fig. 4

##### Etymology.

The specific epithet refers to the type locality.

In Aspergillus subgenus Circumdati section Nigri series *Japonici*.

##### Typification.

China • Yunnan Province, Xishuangbanna Dai Autonomous Prefecture, Mengla County, Menglun Town, Xishuangbanna Tropical Botanical Garden, Chinese Academy of Sciences, Green Stone Forest, 21°54'39"N, 101°17'00"E, in soil of limestone seasonal rainforest, 28 May 2024, Zhao-Qing Zeng, culture, Xiao Mou, ZYN05-01 (holotype HMAS 354081, ex-type strain CGMCC 3.29152).

**Figure 4.**
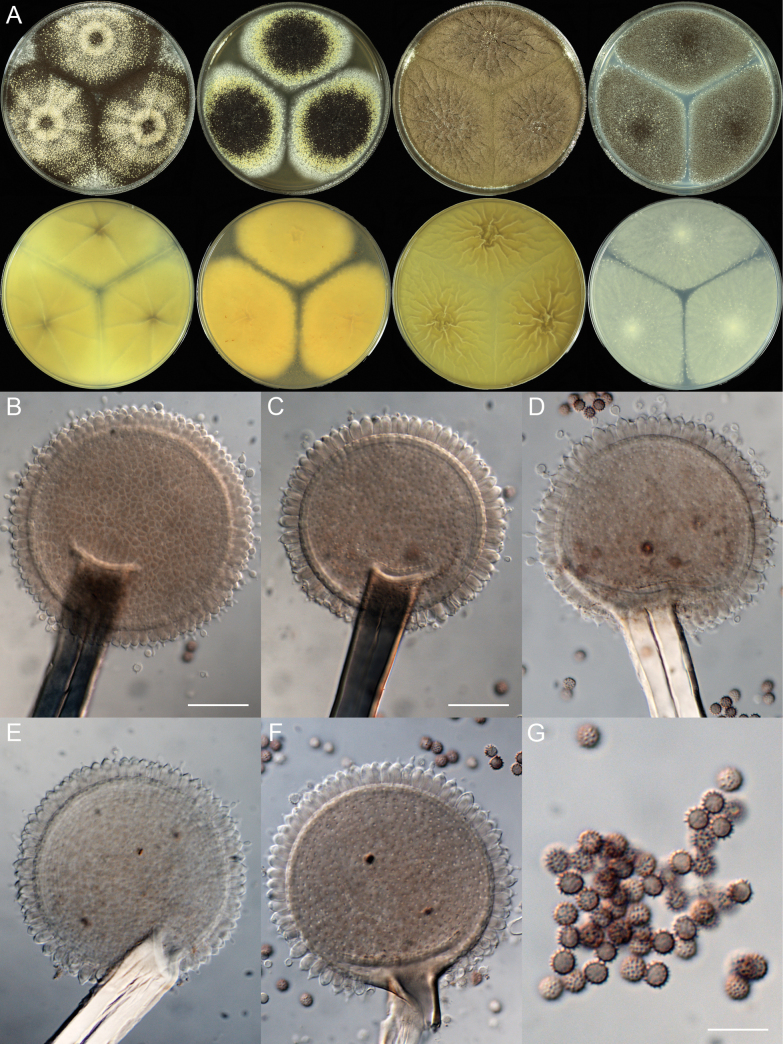
*Aspergillus xishuangbannaensis* (ZYN05-01). A. Colonies: top row left to right, obverse CYA, MEA, YES, and PDA; bottom row left to right, reverse CYA, MEA, YES, and PDA; B–F. Conidiophores; G. Conidia. Scale bars: 22.5 µm (B); 20 µm (C–F); 10 µm (G).

##### DNA barcodes.

ITSPV883252, BenAPV877069, CaMPV877072, RPB2PV877075.

##### Colony diam.

7 days, 25 °C (unless stated otherwise): CYA 64–70 mm; CYA 37 °C 35–57 mm; MEA 55–60 mm; YES 68–70 mm; PDA 66–70 mm.

##### Colony characters.

CYA 25 °C, 7 d: Colonies deep, plane, radially sulcate; margins entire; mycelium white; texture velutinous; sporulation moderately dense; conidia *en masse* dark brown; soluble pigments absent; exudates absent; sclerotia yellow, abundant; reverse yellow brown.

CYA 37 °C, 7 d: Colonies deep, protuberant, radially sulcate; margins narrow, entire; mycelium white; texture velutinous; sporulation dense; conidia *en masse* dark brown; soluble pigments absent; exudates tiny; reverse yellow brown to greyish black.

MEA 25 °C, 7 d: Colonies plane; margins moderately wide, entire; mycelium white; texture velutinous; sporulation dense; conidia *en masse* blackish brown; soluble pigments absent; exudates absent; sclerotia yellow, abundant; reverse yellow brown.

YES 25 °C, 7 d: Colonies deep, radially sulcate; margins entire; mycelium white; texture velutinous; sporulation very dense; conidia *en masse* purplish brown; soluble pigments absent; exudates absent; sclerotia yellow or white; reverse yellow brown.

PDA 25 °C, 7 d: Colonies plain; margins narrow, entire; mycelium white; texture velutinous; sporulation dense; conidia *en masse* dark brown; soluble pigments absent; exudates absent; sclerotia yellow; reverse white.

##### Micromorphology.

Conidial heads radiate; stipes 400–975 × 12–21.5 µm, not septate, walls thick, smooth, hyaline, brownish or black; vesicles 62–80 × 60.5–77.5 µm, globose or subglobose; uniseriate; phialides 6.5–8.5 × 3.5–5.5 µm, flask-shaped and cover the entire surface of the vesicle; conidia 4.5–5.0 × 4.0–5.0 µm, subglobose, echinulate, light brown to dark brown when mature.

##### Notes.

*Aspergillus xishuangbannaensis* is molecularly and morphologically differentiated from its closely related sisters: *A. japonicus*, *A. uvarum*, and *A. indologenus*. For the BenA gene, it differs from *A. japonicus* by 13 bp (97.27% sequence identity), from *A. uvarum* by 16 bp (96.26%), and from *A. indologenus* by 14 bp (97.06%); for the CaM region, it differs from *A. japonicus* by 20 bp (95.97%), from *A. uvarum* by 15 bp (96.98%), and from *A. indologenus* by 17 bp (96.72%); and for the RPB2 fragment, it differs from *A. japonicus* by five bp (99.50%), from *A. uvarum* by 19 bp (98.19%), and from *A. indologenus* by seven bp (99.33%). Although species of this series are similar in gross morphology, the new species can be easily separated from its sisters by much larger vesicles (Table 4). Additionally, sclerotia were not observed in *A. indologenus*, while white to cream sclerotia were often produced by *A. japonicus*, yellow ones in the new species, and dark brown to black ones in *A. uvarum* (Klich 2002; Samson et al. 2007; Perrone et al. 2008; Varga et al. 2011).

## ﻿Discussion

In our previous study, four new species of *Aspergillus* were described from the Xisha Islands in the South China Sea (Wang and Zhuang 2022). That was the first report of a new species of the genus from Chinese tropical islands, suggesting that the biodiversity level of *Aspergillus* might be underestimated in marine environments. *Aspergillus liaoningensis*, *A. plumeriae*, and *A. subinflatus* were later found in tidal flat sediments (Liu et al. 2023), and *A. halopiscium* occurred in the dried marine anchovy *Stolephorus commersonnii* Lacepède (Crous et al. 2025). These accumulated findings reveal that marine environments are biodiversity hotspots for the genus.

In contrast, the two new species discovered in this study are both from terrestrial ecosystems, including a cultural heritage site and a nature reserve. This suggests that terrestrial ecosystems also should not be overlooked in biodiversity investigations.

The Xiangtangshan Caves, located in the southern part of Hebei Province in North China, are a famous cultural treasure containing 16 caves adorned with over 4,000 Buddhist sculptures dating back more than 1,400 years. The new species *A. hebeiensis* was isolated from a soil sample collected in this area. Chinese Buddhist temples have been shown to play an important role in preserving regional biodiversity (Wang et al. 2020; Huang et al. 2025). Further explorations in these traditional cultural and religious areas should be emphasized.

Caves are another biodiversity hotspot, and several *Aspergillus* species were originally reported from them. *Aspergillus alvaroi* was isolated from sediment in a Brazilian cave, *A. guanovespertilionum* from hematophagous bat guano (Lima et al. 2024), *A. lebretii* V.C.S. Alves et al. from cave air (Alves et al. 2022), *A. okavangoensis* Visagie & Nkwe from bat guano-contaminated soil in a Botswana cave (Visagie et al. 2021), and *A. limoniformis* Z.F. Zhang & L. Cai, *A. phialiformis* Z.F. Zhang & L. Cai, and *A. phialosimplex* Z.F. Zhang & L. Cai from dung of Chiroptera, rock, and plant debris in Chinese caves, respectively (Zhang et al. 2021).

The tropical seasonal rain forest in Xishuangbanna is one of the most species-rich forest ecosystems in China. This area is also one of the biodiversity hotspots in the world. Xishuangbanna National Nature Reserve is one of China’s first nature reserves, established in 1958. It consists of five geographically disconnected sub-reserves: Mangao Reserve, Mengla Reserve, Menglun Reserve, Mengyang Reserve, and Shangyong Reserve. Xishuangbanna Tropical Botanical Garden, located in Menglun Town and covering 1,125 hectares, is home to more than 14,000 plant species, including rare orchids and ancient cycads. Many fungal species were first described from Xishuangbanna, e.g., *Chloridium xishuangbannaense* W.P. Wu & Y.Z. Diao (Wu and Diao 2022), *Talaromyces bannicus* L. Wang (Wei et al. 2021), and *Trichoderma bannaense* Kai Chen & W.Y. Zhuang (Chen and Zhuang 2017). However, no new species of Aspergillaceae had been recorded from this district. *Aspergillus xishuangbannaensis*, described here, was isolated from a soil sample collected in this area. More surveys are urgently needed to explore the species diversity of this group in diverse terrestrial ecosystems in China.

## Supplementary Material

XML Treatment for Aspergillus hebeiensis

XML Treatment for Aspergillus xishuangbannaensis

## ﻿Additional information

### Use of AI

No use of AI was reported.

### Author ORCIDs

Lu-Yao Peng https://orcid.org/0009-0004-9179-4826

Wen-Ying Zhuang https://orcid.org/0000-0002-1162-9678

Xin-Cun Wang https://orcid.org/0000-0003-1780-7735

### Data availability

All of the data that support the findings of this study are available in the main text or Supplementary Information.

## Supplementary materials

Supplementary material 1 Мaximum likelihood phylogenies Data type zip https://binary.pensoft.net/file/1456798This dataset is made available under the Open Database License (http://opendatacommons.org/licenses/odbl/1.0/). The Open Database License (ODbL) is a license agreement intended to allow users to freely share, modify, and use this Dataset while maintaining this same freedom for others, provided that the original source and author(s) are credited.Lu-Yao Peng, Wen-Ying Zhuang, Xin-Cun Wang
