# Supplementary material for: ﻿New species of Aspergillus in sections Cavernicolarum and Nigri from terrestrial ecosystems of China (Eurotiales, Aspergillaceae)
Source: MycoKeys. 2025 Nov 3;124:275–90. doi: 10.3897/mycokeys.124.172775 (PMC12603649; doi:10.3897/mycokeys.124.172775)
Supplement: Supplementary material 1 — Мaximum likelihood phylogenies [file mycokeys-124-275-s001.zip › Supplementary/Supplementary Legends.docx]

**Supplementary Legends**

Figure S1. Maximum likelihood phylogeny of *Aspergillus* subgenus *Nidulantes* section *Cavernicolarum* inferred from ITS dataset. Bootstrap values ≥ 70% are indicated at nodes. Asterisk denotes 100% bootstrap.

Figure S2. Maximum likelihood phylogeny of *Aspergillus* subgenus *Nidulantes* section *Cavernicolarum* inferred from BenA dataset. Bootstrap values ≥ 70% are indicated at nodes. Asterisk denotes 100% bootstrap.

Figure S3. Maximum likelihood phylogeny of *Aspergillus* subgenus *Nidulantes* section *Cavernicolarum* inferred from CaM dataset. Bootstrap values ≥ 70% are indicated at nodes. Asterisk denotes 100% bootstrap.

Figure S4. Maximum likelihood phylogeny of *Aspergillus* subgenus *Nidulantes* section *Cavernicolarum* inferred from RPB2 dataset. Bootstrap values ≥ 70% are indicated at nodes. Asterisk denotes 100% bootstrap.

Figure S5. Maximum likelihood phylogeny of *Aspergillus* subgenus *Circumdati* section *Nigri* series *Japonici* inferred from BenA dataset. Bootstrap values ≥ 70% are indicated at nodes. Asterisk denotes 100% bootstrap.

Figure S6. Maximum likelihood phylogeny of *Aspergillus* subgenus *Circumdati* section *Nigri* series *Japonici* inferred from CaM dataset. Bootstrap values ≥ 70% are indicated at nodes. Asterisk denotes 100% bootstrap.

Figure S7. Maximum likelihood phylogeny of *Aspergillus* subgenus *Circumdati* section *Nigri* series *Japonici* inferred from RPB2 dataset. Bootstrap values ≥ 70% are indicated at nodes. Asterisk denotes 100% bootstrap.
