# Supplementary figures and images for: ﻿New species of Aspergillus in sections Cavernicolarum and Nigri from terrestrial ecosystems of China (Eurotiales, Aspergillaceae)
Source: MycoKeys. 2025 Nov 3;124:275–90. doi: 10.3897/mycokeys.124.172775 (PMC12603649; doi:10.3897/mycokeys.124.172775)

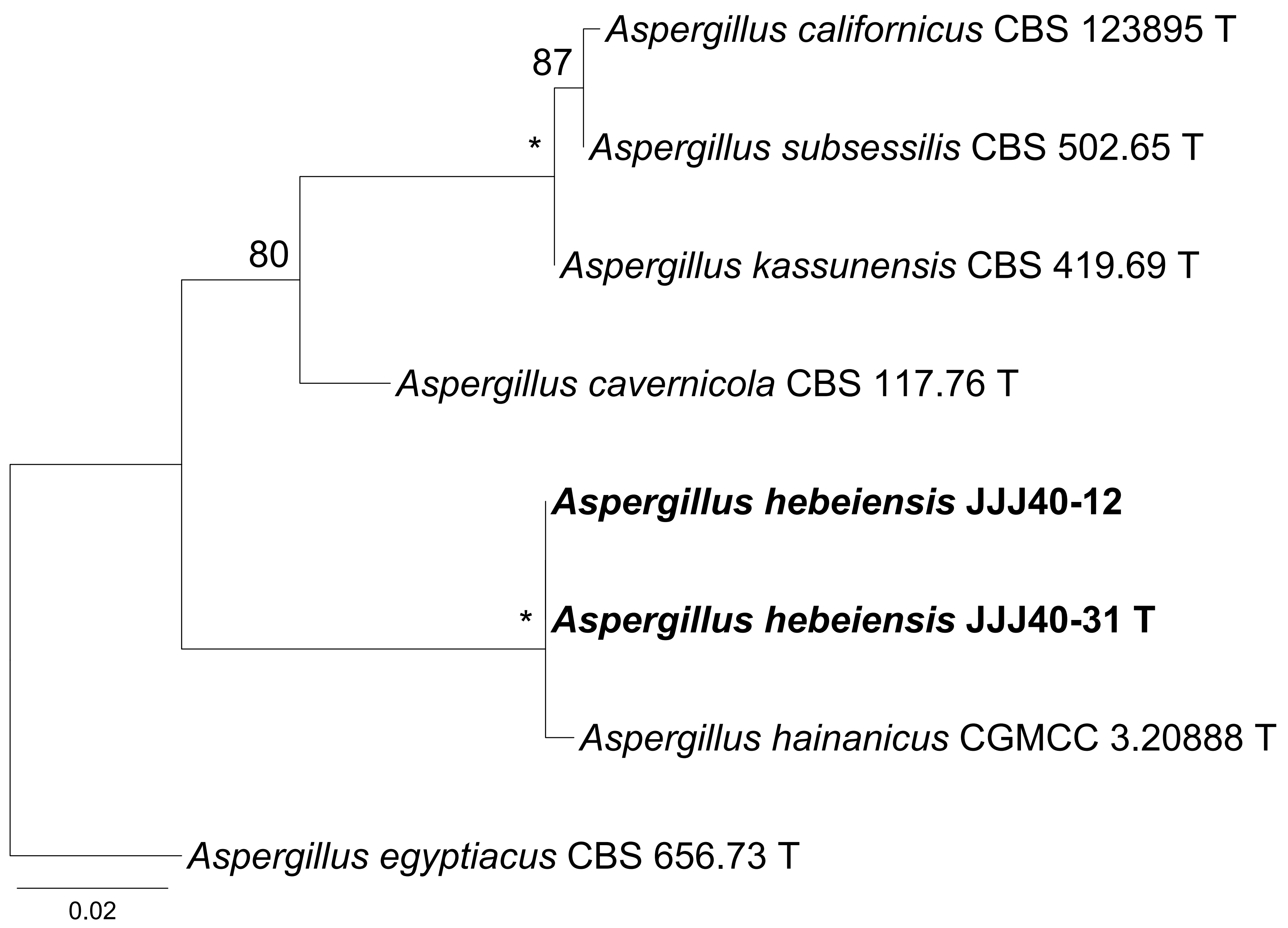

Supplement: Supplementary material 1 — Мaximum likelihood phylogenies [file mycokeys-124-275-s001.zip › Supplementary/Figure S1 Caver ITS.pdf]

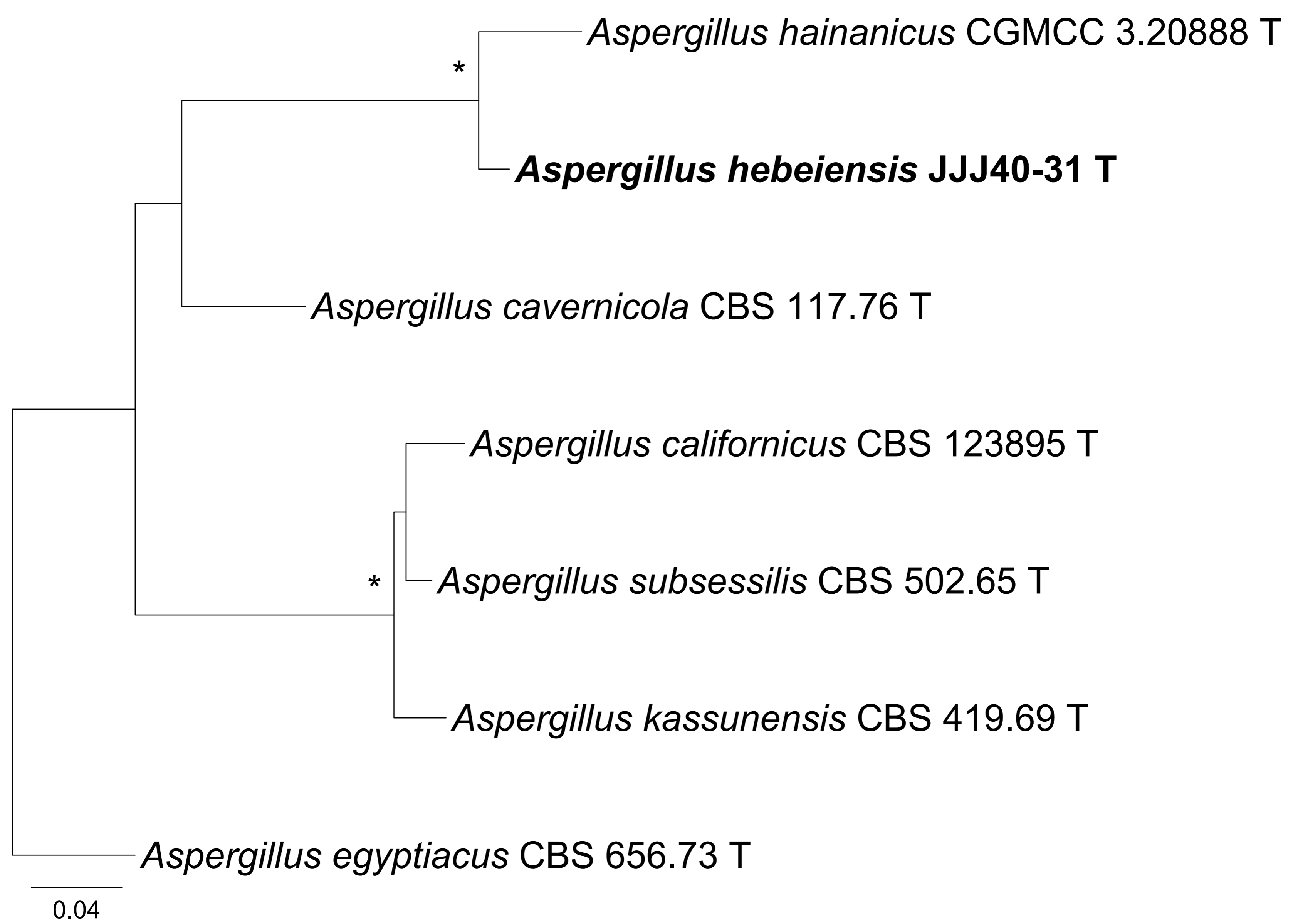

Supplement: Supplementary material 1 — Мaximum likelihood phylogenies [file mycokeys-124-275-s001.zip › Supplementary/Figure S2 Caver BenA.pdf]

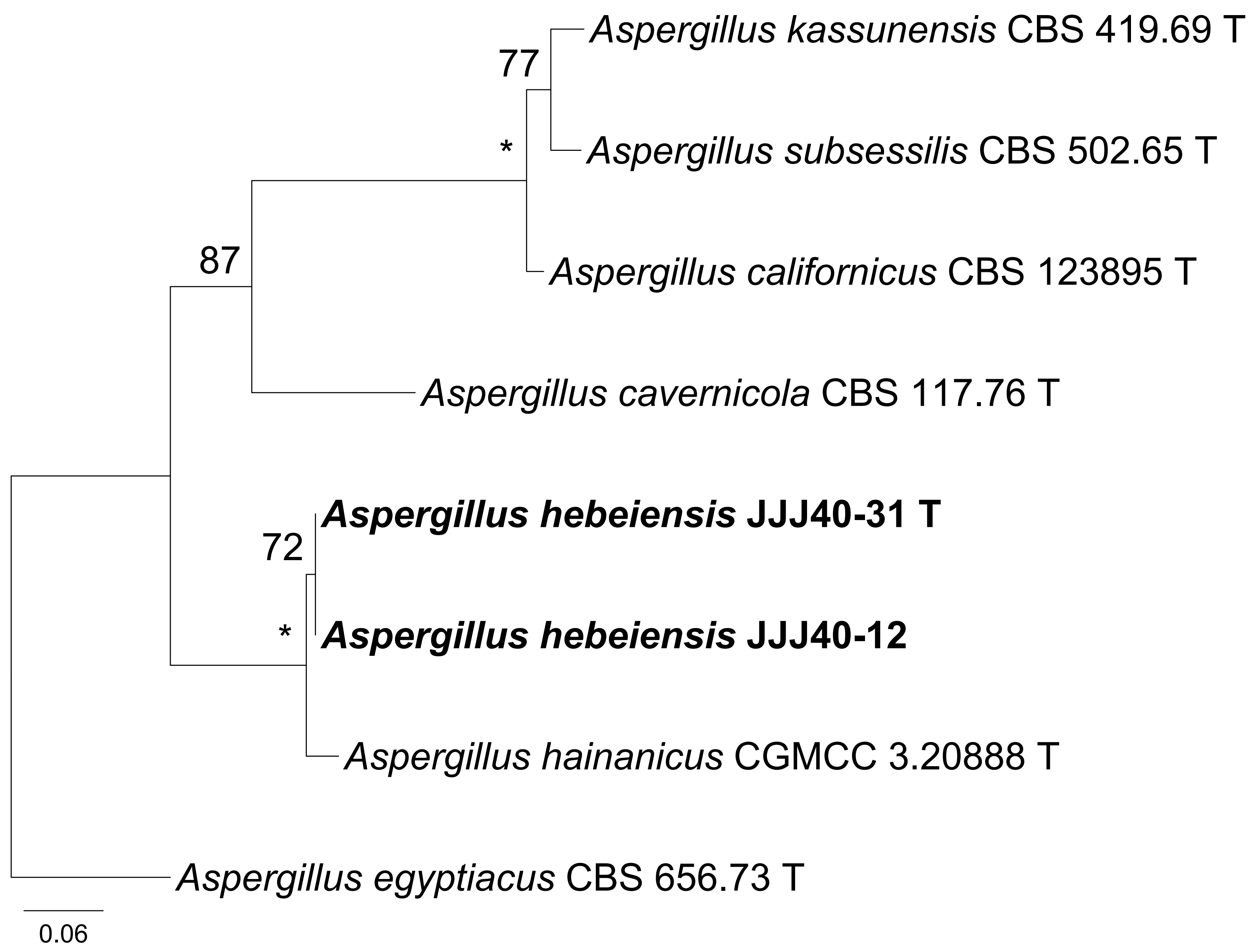

Supplement: Supplementary material 1 — Мaximum likelihood phylogenies [file mycokeys-124-275-s001.zip › Supplementary/Figure S3 Caver CaM.pdf]

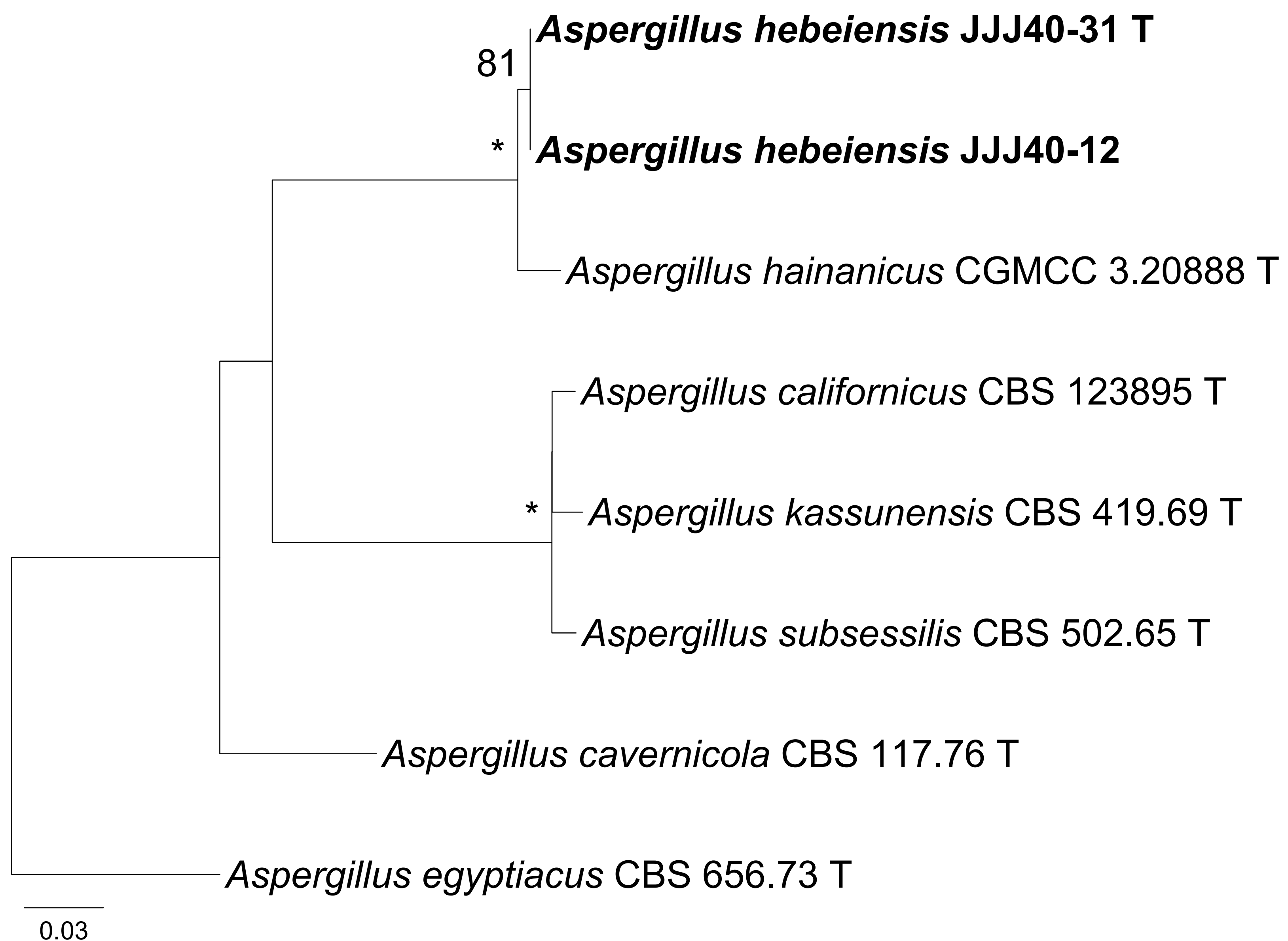

Supplement: Supplementary material 1 — Мaximum likelihood phylogenies [file mycokeys-124-275-s001.zip › Supplementary/Figure S4 Caver RPB2.pdf]

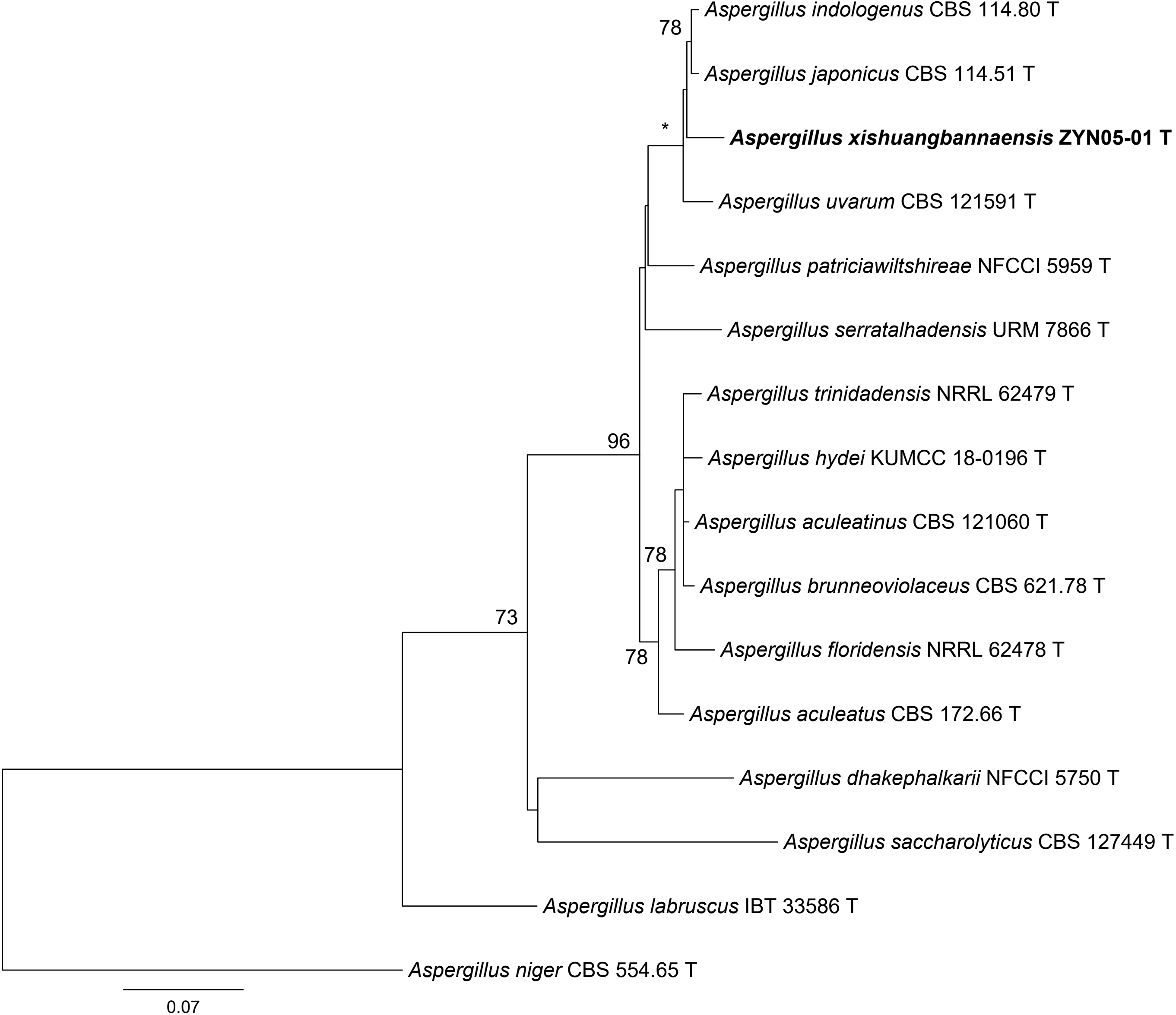

Supplement: Supplementary material 1 — Мaximum likelihood phylogenies [file mycokeys-124-275-s001.zip › Supplementary/Figure S5 Japonici BenA.pdf]

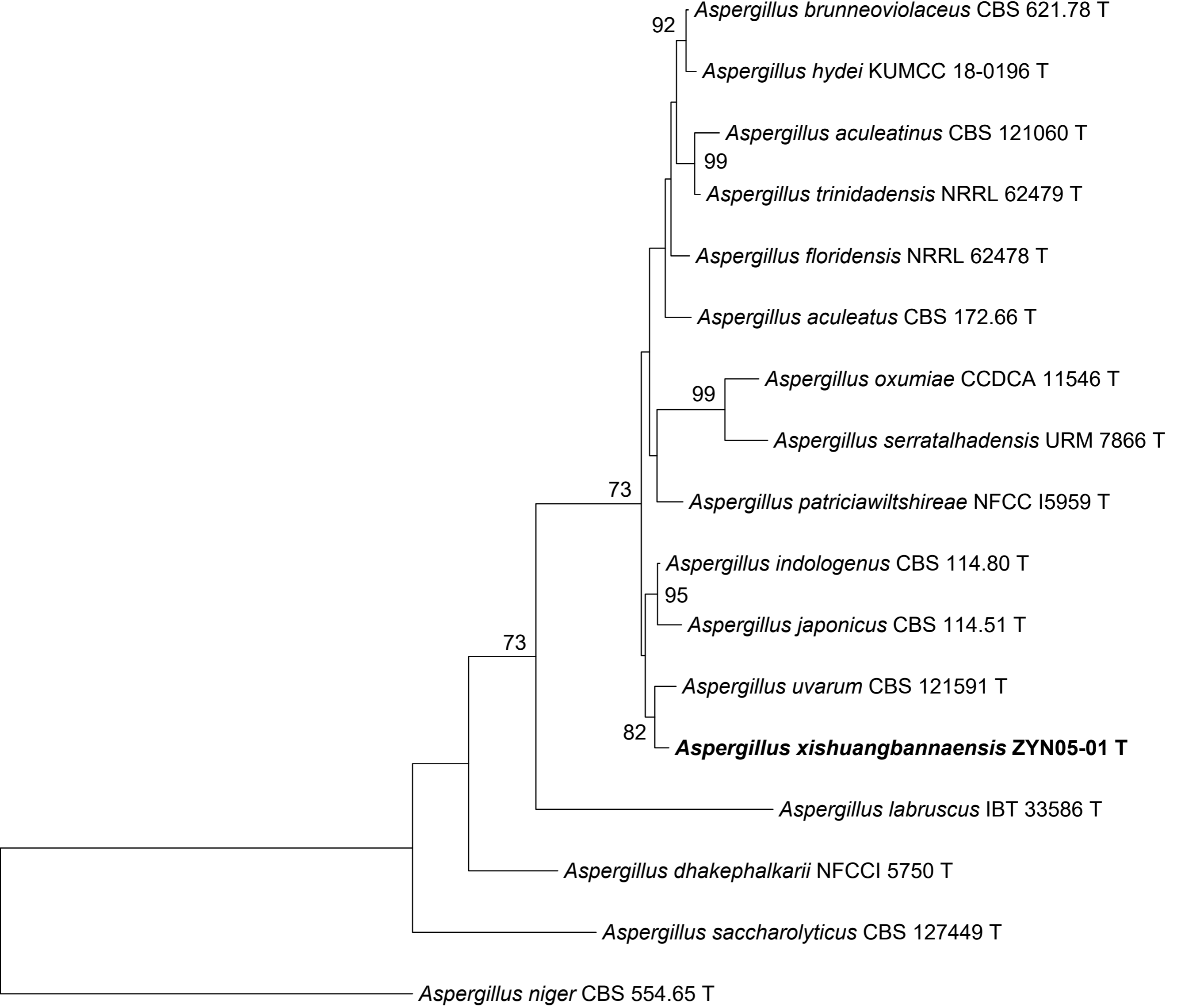

Supplement: Supplementary material 1 — Мaximum likelihood phylogenies [file mycokeys-124-275-s001.zip › Supplementary/Figure S6 Japonici CaM.pdf]

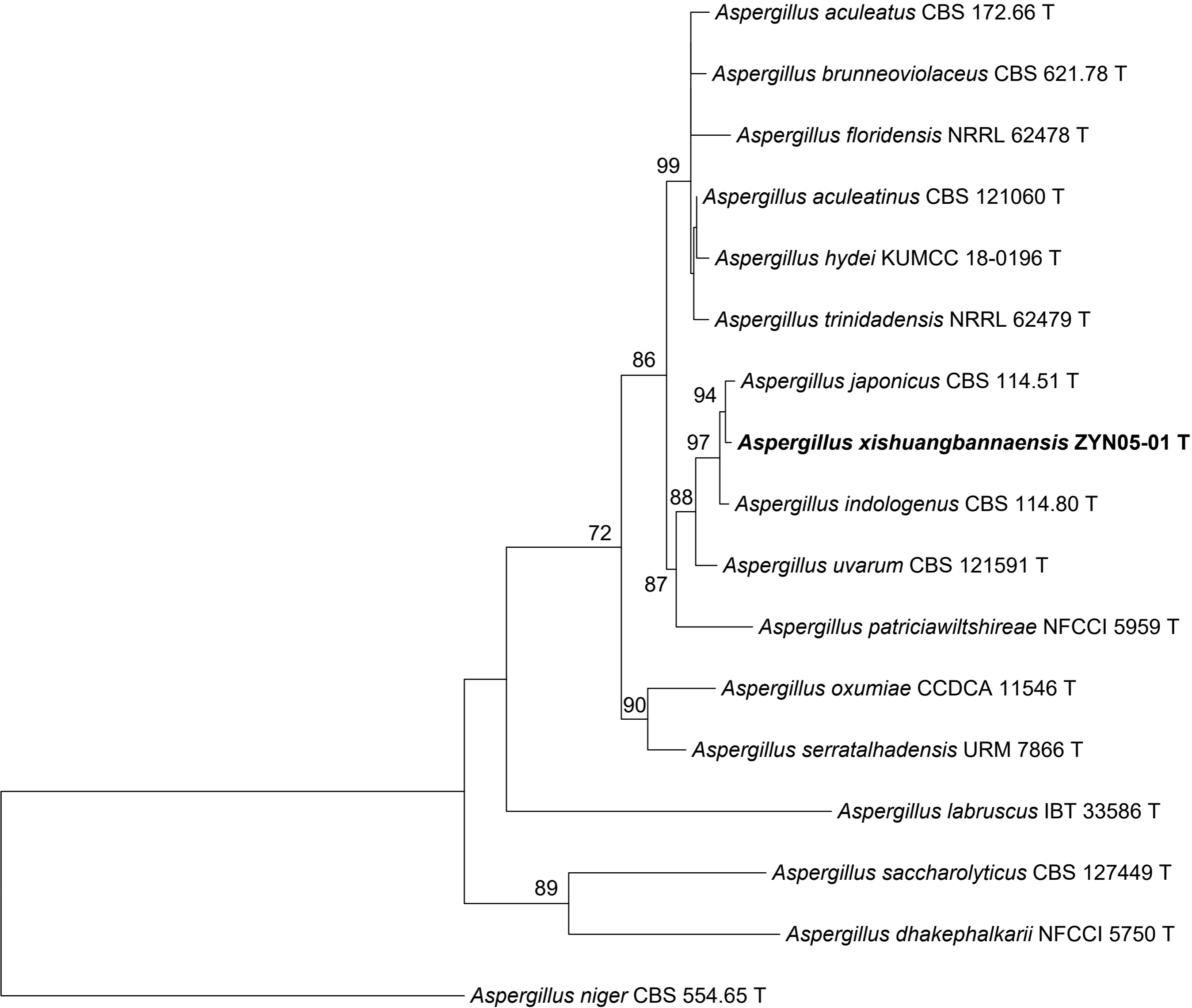

0.04

Supplement: Supplementary material 1 — Мaximum likelihood phylogenies [file mycokeys-124-275-s001.zip › Supplementary/Figure S7 Japonici RPB2.pdf]
